# Impact of frozen storage on some functional properties and sensory evaluation of goose meat

**DOI:** 10.1016/j.psj.2023.102894

**Published:** 2023-06-29

**Authors:** M. Wereńska, A. Okruszek

**Affiliations:** Department of Food Technology and Nutrition, Wroclaw University of Economics and Business, Wroclaw 53-345, Poland

**Keywords:** goose meat, frozen storage, functional property, sensory evaluation, CIE Lab

## Abstract

The objective of this study was to investigate the changes in the functional properties (pH, water holding capacity [**WHC**], water binding capacity [**WBC**], cooking losses [**CL**], defrosting losses [**DL**]), color parameters (L*, a*, b*, C, h°, ΔE), and sensory evaluation of breast (**BM**) and leg (**LM**) muscles from 17-wk-old female White Kołuda geese packaged in a vacuum and stored in frozen conditions at −20°C. During 17 wk, the geese were fed ad libitum on the same complete feed. The samples (18 BM and 18 LM) from the right part of the carcasses were stored for 30, 90, 80, 270, and 365 d. The changes in functional properties were established using a standard method used in the meat industry, according to Wierbicki et al. (1962), Grau and Hamm (1953), and CIE, (1986). Sensory evaluation was established according to defined parameters in PN-ISO 8586-2:2008. The time of frozen storage affected the decrease in WHC and WBC of BM and LM. Moreover, the LM can be characterized by a higher WHC and WBC compared to the values in the BM. It was established that CL and DL, which are the critical quality indicators, negatively increased in BM and LM during frozen storage. Considering the sensory evaluation and L*, a*, b*, C, it was established that changes in BM and LM during frozen storage were unfavorable. The scores given for smell, taste, consistency, and general appearance, as also L*, a*, and b* parameters decreased significantly during frozen storage. In addition, BM received lower scores for general appearance (at 180th and 270th day), and L* (in all frozen storage), than LM. BM and LM characterized the parameter ΔE in the range of 0.44 to 1.45, which allowed us to conclude that slight color differences were visible in these muscles (<2). Based on the study, it can be suggested that the optimal frozen storage time for BM and LM should not be longer than 180 d.

## INTRODUCTION

The meat industry and meat processing occupy an important place in the world. Meat consumption trends vary from region to region across the world. While in some parts of the world, meat consumption might be increasing, in others, it might be decreasing (Suleman et al., 2019). Food and Agriculture Organization of the United Nations (**FAO**) data indicate that generally meat consumption will be increase, and in 2023, the poultry meat production will be about 361 million tones, and in 2029 will be around 373 million tones (https://fao.org). Global meat production is increasing due to population growth and their needs. Waterfowl production has been on an upward trend for many years and has become increasingly important around the world (Huang et al., 2012; Gornowicz and Lewko, 2016). According to statistics, the total goose production in Poland was 1.026 thousand carcasses and it was about 20 thousand tons of geese meat (GUS, 2020). In 2020, in the world, there were 1.15 billion ducks, and total goose and guinea fowl meat production in 2020 reached 364 million birds (www.fao.org/poultry-production-products). For centuries, meat and its products have been essential components of our diet, providing a major proportion of consumer requirements for amino acids, fatty acids, some vitamins and minerals. Goose meat, among others, is very favorable from a nutritional point of view. It contains all the essential amino acids and the highest amount of unsaturated fatty acids among all kinds of meat (Boz et al., 2019; Wereńska et al., 2021). The traditional method of fattening birds for oat grain results in the formation of fat in goose meat with a higher content of valuable polyunsaturated fatty acids (**PUFAs**) (Uhlíová et al., 2018; Biesek et al., 2020). Furthermore, waterfowl fat is considered to be safe for consumers due to its relatively low level of saturated fatty acids. In addition, goose meat is a valuable source of protein (about 20%), amino acids, including essential ones, as well as vitamins and minerals such as zinc, phosphorus, magnesium, sodium, calcium, potassium and iron (Goluch and Pilarczyk, 2022; Goluch and Haraf, 2023; Wereńska et al., 2023).

The quality of meat obtained from slaughtered animals, including waterfowl, is determined primarily by its nutritional value, health safety, consumer acceptance (associated with sensory attributes), and technological suitability determined by functional properties. Muscle proteins, mainly myofibrillar proteins, play an important role in shaping the functional properties of raw meat. They affect the ability of meat to water holding capacity, which is one of the critical factors determining the amount of cooking and defrosting losses (Hughes et al., 2014). The results of the tests on poultry meat indicate that the functional properties deteriorate with the extension of the frozen storage time (Śmiecińska et al., 2015; Ablikim et al., 2016; Wei et al., 2017). The pH value determines meat's technological suitability (Tong et al., 2014). Changes in the pH value of meat during its frozen storage are influenced, among others, by the freeze denaturation of proteins contained in it. Changes in the pH value that occurred during frozen storage affected the functional properties of meat, including the ability to water holding, and water binding capacity, as well as cooking and defrosting losses (Rahman et al., 2014; Alonso et al., 2016). It should be taken into account that the value of water holding capacity (**WHC**) and water binding capacity (**WBC**) may also result from physiological and genetic differences, pH of meat, and the amount of protein. The age of the geese, type of muscles, degree of fattening, and amount of connective tissue also affect WBC and WHC. Moreover, the meat of young birds, which contains more the amount of procollagen, is characterized by a better ability to hold its own water than old bird meat. According to Vieira et al. (2009), the reason for the differences in WHC in chilled and frozen meat could be related to the disruption of the muscle cell structure upon freezing. On the other hand, Farouk et al. (2004) have reported higher drip in slowly frozen meat than in fast frozen meat, which was associated with a greater structural damage caused by larger intracellular ice crystals produced during slow freezing. The size of the defrosting losses of course also determines the technological properties (Sales et al., 2020). It depends primarily on the type of muscle, the method of freezing the raw material, the conditions of its frozen storage, and the method of thawing (Aidani et al., 2014; Cho et al., 2017). The amount of thawing drip from meat results from irreversible damage to the histological structure of muscle tissue during its freezing (Vieira et al., 2009; Leygonie et al., 2012a; Traore et al., 2012). The consequence of increased DL (defrosting losses) is the loss of many valuable nutrients in meat. During defrosting, meat losses nonprotein nitrogen compounds, minerals, and vitamins (Śmiecińska et al., 2015; Falowo et al., 2017). The increased volume of the defrosting losses, similar to the cooking losses, also affects the deterioration of sensory attributes of meat, mainly its juiciness (Vieira et al., 2009; Akhtar et al., 2013; Augustyńska-Prejsnar et al., 2021). The thawed material may have a reduced nutritional value and technological usefulness compared to unfrozen ones. The color of the meat is an important quality distinguishing feature, classified as external, critical sensory attributes (Font-i-Furnols and Guerrero, 2014). The color of the meat is shaped by its physical structure and the overall content of heme pigments. Important is the relationship of myoglobin, oxymyoglobin, and metmyoglobin content (Faustman et al., 2010; Suman and Joseph, 2013). The color of the meat changes during its frozen storage. The presence of individual forms of heme pigments in the skeletal muscle tissue of animals is reflected in the instrumentally measured parameters of the color of raw meat (Zakaria et al., 2015). Oxidation of heme pigments increases with the extension of the frozen storage time, contributes primarily to the reduction of the photometric lightness and the intensity of the red color of the meat. The oxidation of myoglobin to metmyoglobin is among others reasons of this phenomenon and formation of the brown color of meat (Muela et al., 2015; Zakaria et al., 2015; Alonso et al., 2016).

Due to the seasonal nature of goose meat production, it is necessary to freeze and store it in a frozen condition. Frozen storage is one of the most significant strategies for preserving food quality during long-term. Unfortunately, as described above, many unfavorable changes occur during frozen storage (Li et al., 2022). Taking the above into account, the aim of our study was to analyze the changes in functional properties and sensory parameters of goose meat during frozen storage. The research results will be of great importance to consumers and producers of this meat.

On the other hand, information on the quality of frozen goose meat is scarce in the literature on the subject. Therefore, this a research gap, the Department of Food Technology and Nutrition employees at the University of Economics in Wrocław meticulously fill.

## MATERIAL AND METHODS

### Meat Samples

The experimental material for each experimental periods (control, 30, 90, 180, 270, and 365 d) consisted of breast muscle (**BM**) (*n* = 18) and leg (thigh) muscles (**LM**) (*n* = 18) from 17-wk-old female White Kołuda geese (W 31). The geese were reared on the same farm and fed with concentrated diet (Wołoszyn et al., 2020). The birds were slaughtered in an industrial slaughterhouse according to EU regulations. The carcasses were bled, scalded (approximately 1 min, at 63°C), plucked, and eviscerated. The eviscerated carcasses were placed immediately inside a refrigerator at 4°C for 24 h. Afterward, the BM and LM were cut out from the right side of the carcasses, and then individually packed in a head shrink bag Supravis SHRINK BAG P. The average weight for BM (with skin and subcutaneous fat) was 465 g ± 21 g, and for LM, it was (with skin and subcutaneous fat) 405 g ± 18 g. The packed muscles were frozen in an air tunnel at −20°C, measured at their geometric center. Then, the muscles were placed in a freezer cabinet (HSA29530N, Beko, Warszawa, Poland) and stored for 30, 90, 180, 270, and 365 d at a temperature of −20°C (±1°C). In each frozen storage time, 18 BM and 18 LM were investigated. Thirty-six (18 breasts + 18 legs) fresh muscles (24 h after the slaughter at +4°C) were used for the control (**C**) group, and results obtained for this group were taken as initial values.

### Sample Preparation

To analyze pH, WHC, WBC, defrosting losses (**DL**), cooking losses (**CL**), CIE Lab, and sensory evaluation, the muscles were thawed in a refrigerated cabinet (LG, M600, Seoul, South Korea) for 24 h at +4°C. Next, for determining the WHC, WBC, and CL, the skin and subcutaneous fat were separated from the muscles. The pH, DL, CIE Lab, and sensory evaluation were determined in intact muscles. Then, each BM and LM has been ground (mesh diameter of 2 mm) in an electric bowl chopper (model MM/1000/887, Zelmer, Rzeszów, Poland) to determine WHC, WBC, and CL.

### Functional Properties

The assessment of the functional properties of the muscles included measurements of the pH value and the determination of the WHC, the WBC, as well as cooking and defrosting losses.

### pH Measurement

The pH measurement in the muscle was performed using a Double Pore Slim combination dagger electrode (Hamilton Robotics, Reno, NV) connected to a CyberScan pH 1500 pH meter (Eutech Instrument, Vernon Hills, IL). The measurement consisted in inserting an electrode into the muscle. The final result was the mean of 3 measurements taken in different locations in the muscle. The measurement was made in 3 repetitions in each goose muscle.

### Water Holding Capacity

Water holding capacity was determined based on the free water mass squeezed from a ground meat sample by Grau and Hamm (1953) method. Ground meat samples (weighing about 300 mg) were placed on a weighed filter paper between 2 glass tiles. A force of 2 kg was applied to each sample. The measurement was made in 3 repetitions in each goose muscle. After 5 min of squeezing, the filter paper with a stain of squeezed meat juice was immediately weighed. WHC was calculated according to the following formula:

WHC=(Z−U)/Z×100[%];

where *Z* is the water content in the sample (mg), *U* is the loss of meat juice due to the applied load (mg).

### Water Binding Capacity

The determination of WBC consists in homogenizing the meat with the appropriate addition of water and then centrifuging the water that has not been bound Wierbicki et al. (1962). Ground meat (20 g) was homogenized (10,000 rpm for 2 min) with 60 cm^3^ distilled water at 25°C. The obtained homogenate was poured into 2 tubes (35 cm^3^ each) and centrifuged for 10 min at 4,000 rpm. The WBC of meat proteins was calculated from the average volume of the obtained clear liquids (ʋ) using the formula:

WBC=300−(11.43×υ)[%]

The measurement was made in 3 repetitions in each goose muscle.

### Defrosting Losses

Defrosting losses were calculated from the weight difference before (W_before_), and after (W_after_) defrosting the muscles. The DL from each muscle was calculated using the following equations:

DL = (W_before_ - W_after_)/W_after_) × 100%

### Cooking Losses

To determine the cooking losses, 20 g of ground meat (with an accuracy of 0.01 g) was weighed (W_before_), shaped into a ball, and placed in a round metal strainer in boiling in a water bath (SW 22, Julabo, GmbH, Seelbach, Germany). The meatballs were cooked for 15 min, then cooled and weighed (W_after_). The CL was calculated using the following equations:

CL = (W_before_ - W_after_)/W_before_) × 100%

The measurement was made in 3 repetitions in each goose muscle.

### Color Measurements

The measurements are presented in the L*, a*, b* color scale (CIE, 1986) using a Minolta chromameter (model CR-310, Konica Minolta Co. Ltd., Osaka, Japan) with an illuminant D_65_ and 8 mm viewing port. The L* parameter signifies the lightness of the color, and it is located on a vertical axis in space, and its value ranges from 0 (black) to 100 (white). The coordinates a* and b* represent the values from which saturation and hue of color can be calculated. The value of 0º for +a* represents red, 90º for +b* yellow, 180º for –a*green and 270º for –b* blue. The C value stands for saturation (chroma), and it has a zero value in the center and increases with the distance from the center, hº is a hue angle expressed in degrees and has its origin on the +a* axis. Before measurement, the apparatus was calibrated according to the white reference standard Y = 94.2; x = 0.313; y = 0.324. The values of L*, a*, and b* were measured across the cut surface of the raw and defrosting meat. The means of the reading on 3 random locations of each muscle were determined. Chroma (C) and hue angle (h^o^) values were obtained from the a*value and b*value following equations (CIE, 1986):

$$C={\left(a{*}^{2}+b{*}^{2}\right)}^{1/2}$$

hº = tg^−1^ (b*/a*), were hº = 0º for reddish hue and hº = 90º for yellowish hue.

Color space CIE L*, a*, b* allows identifying, count and measure objective variances between the different colors. This difference, consisting of deviations ΔL*, Δa*, Δb*, is best expressed by the term ΔE, which is a square root of the sum of the individual deviation squared. The individual differences (ΔE) in L*, a* and b* values were calculated from the formula ΔE=[(ΔL*)^2^ + (Δa*)^2^ + (Δb*)^2^]^1/2^ (CIE, 1986).

ΔE was calculated as the difference between consecutive storage periods.

### Sensory Evaluation

The sensory evaluation of control sample and defrosting muscles was carried out by a trained, 9-person team with established and proven sensory sensitivity (defined based on the following standards PN EN ISO 8586, 2014) with previous experience with sensory analysis of meat. All sensory work was carried out at the sensory laboratory in the Department of Food Technology and Nutrition in Wroclaw University of Economics and Business (Poland). The sensory laboratory was prepared to work following guidelines give in EN ISO 8589 (2010). Initially, the panel members agreed on the descriptors (general appearance, color, smell, consistency). Samples for evaluation were breast or leg muscles cut out from the right part of carcasses. They were served on a white tray, coded with 3-digit codes and served to the sensory panel for analysis. The sensory evaluation of raw muscles was carried out immediately after thawing, using the 6-point CU (contractual unit) scale according to the specifications given in Table 1.

**Table 1:** Sensory evaluation of raw BM and LM of the White Kołuda geese.

	Score [CU]	Sensory descriptors			
		Color	Consistency	Smell	General appearance
Unacceptable	**1**	Completely changed, gray, brown, or blue	The muscle tissue is relaxed and easily separates when pressed	Very heavily changed, putrefactive	Completely changed, surface: sticky/slimy
	**2**	Heavily altered, brown-brown/dark brown	The muscle tissue is relaxed and flattens out when pressed	Heavily changed, sour	Heavily altered, surface: slightly sticky/dry
	**3**	Noticeably changed in places, lighter/darker	Muscle tissue deforms permanently after pressure with a finger	Slightly changed	Noticeably changed, surface: dry/slightly sticky
Acceptable	**4**	Dark red	Medium elastic, after pressing the deformation is hardly equalized	Poorly changed	Noticeably altered, surface: slightly damp/dry
	**5**	Dark red, even	Muscle tissue elastic, less compact	Typical	Typical, surface: slightly damp/dry
	**6**	Intense dark red, even, typical	Elastic muscle tissue	Perfect, typical	Ideal, typical, wet surface

Abbreviations: BM, breast muscles; LM, leg muscles.

### Statistical Analysis

The data were analyzed as a completely randomized design using a 2-way ANOVA concerning the kind of muscles (breast and leg) and time its frozen storage (30, 90, 180, 270, and 360 d) as a factorial design (2 × 5), according to the following linear model: *Y_ij_* = *μ* + *A_i_* + *B_j_* + (*AB*)*_ij_* + *e_ij_*, where *Y_ij_* = value of trait (the dependent variable); *μ* = overall mean; *A_j_* = effect of kind of muscle; *B_j_* = effect of time frozen storage of muscles; (*AB*) = interaction, and *e_ij_* = random observation error, using Statistica13.3 software (Statistica, 2019). The statistical significance of the differences between the averages of the groups was calculated using Tukey's test and was at a level of *P* ≤ 0.05. The tables present the average values and their standard deviations in Table 2, Table 3, Table 4.

## RESULTS AND DISCUSSION

### Functional Properties

In the literature, we can find information that the pH of geese breast muscles (depend on kind) is at the level of 5.65 to 6.20 (Gardzielewska et al., 2003; Biesiada-Drzazga, 2006; Geldenhuys et al., 2013; Oz and Celik, 2015; Haraf et al., 2023). These values are in agreement with the results of own research, where the pH value was in the range of 5.98 to 5.99. It was shown that the pH of geese muscles increased with the extension of their frozen storage. Significant pH differences (*P* ≤ 0.05) in LM occurred between the 30th and 270th day and in BM between the 30th and 270th to 365th day of frozen storage (Table 2). The pH values of goose meat stored in freezing condition obtained in our research are consistent with the results of (Gardzielewska et al., 2003; Ali and Zahran, 2010; Selani et al., 2011; Śmiecińska et al., 2015). The authors stated an increased pH value of turkey breast and chicken leg muscles with extended frozen storage (from 1.5 to 9 months). On the other hand, Kim et al. (2011), Leygonie et al. (2012b), and Muela et al. (2015) showed the pH value of African ostrich (Struthio camelus) and lamb meat decreased over time of frozen storage. Enzymes naturally present in meat, such as cathepsins and calpains, remain active even at low temperatures during frozen storage. These enzymes have proteolytic activity, meaning they break down proteins. They target the muscle proteins in meat and initiate their degradation. The breakdown of muscle proteins during frozen storage leads to the release of amino acids, including histidine, glutamate, and aspartate. Histidine, in particular, plays a significant role in the increase of pH. Histidine can undergo a process called decarboxylation under certain conditions, including the alkaline environment created by the protein breakdown. Histidine decarboxylation results in the production of histamine and the release of a proton (H+), which increases the pH of the meat (Leygonie et al. 2012a). Waritthitham et al. (2010) and Ablikim et al. (2016) explained the increase in pH by the progressive freezing denaturation of muscle myofibrillar proteins and loss of meat juice. This contributes to an increase in concentration of acidic products. In turn, acidic products result in autolytic changes in muscle tissue. Significant differences (*P* ≤ 0.05) in pH values occurred between the types of muscles. It was shown that the BM were characterized by lower (*P* ≤ 0.05) pH values in individual storage periods (except for the 365th day), compared to the LM (Table 2).

**Table 2:** Functional properties of the BM and LM of the White Kołuda geese.

		Control group	Time of frozen storage (d)				
		C	30	90	180	270	365
Parameters	Type of muscle	*n* = 18					
pH	BM	5.99^cd^ ± 0.08	5.99^cd^ ± 0.07	6.06^bc^ ± 0.05	6.07^bc^ ± 0.07	6.11^b^^,^^y^ ± 0.06	6.19^a^ ± 0.06
	LM	5.98^c^ ± 0.04	6.03^bc^ ± 0.05	6.05^b^ ± 0.04	6.09^b^ ± 0.05	6.17^a^^,^^x^ ± 0.06	6.18^a^ ± 0.05
Water holding capacity (WHC) [%]	BM	60.17^a^^,^^y^ ± 1.71	58.52^a^^,^^y^ ± 1.83	57.86^a^ ± 1.13	54.82^b^^,^^y^ ± 2.09	54.64^b^^,^^y^ ± 2.22	51.12^c^^,^^y^ ± 2.39
	LM	65.46^a^^,^^x^ ± 1.14	63.22^b^^,^^x^ ± 1.17	58.47^c^ ± 1.16	58.31^c^^,^^x^ ± 1.68	57.25^c^^,^^x^ ± 1.85	56.56^c^^,^^x^ ± 1.31
Water binding capacity (WBC) [%]	BM	88.64^a^^,^^y^ ± 2.84	82.32^b^^,^^y^ ± 4.34	^y^77.21^b^^,^^y^ ± 3.35	73.56^c^^,^^y^ ± 3.71	70.28^c^^,^^y^ ± 2.96	67.38^d^^,^^y^ ± 2.59
	LM	93.85^a^^,^^x^ ± 3.41	89.41^b^^,^^x^ ± 3.75	^x^87.02^b^^,^^x^ ± 2.53	83.54^c^^,^^x^ ± 3.01	80.43^d^^,^^x^ ± 2.21	79.37^d^^,^^x^ ± 3.68
Defrosting losses (DL) [%]	BM	-	0.87^c^ ± 0.22	1.15^b^ ± 0.18	1.36^b^ ± 0.20	1.60^a^ ± 0.41	1.87^a^^,^^x^ ± 0.28
	LM	-	0.79^c^ ± 0.21	1.04^b^ ± 0.20	1.21^b^ ± 0.23	1.46^a^ ± 0.21	1.54^a^^,^^y^ ± 0.24
Cooking losses (CL) [%]	BM	21.56^d^ ± 0.95	21.95^d^ ± 1.21	23.94^c^ ± 1.02	25.02^b^^,^^x^ ± 0.96	26.13^a^ ± 0.83	26.65^a^ ± 0.84
	LM	21.22^c^ ± 0.93	21.65^c^ ± 1.08	23.02^b^ ± 1.16	23.47^b^^,^^y^ ± 1.26	25.06^a^ ± 1.20	25.74^a^ ± 1.05

Abbreviations: BM, breast muscles; LM, leg muscles.

a-d Different letters in a row mean statistically significant differences between group average, including storage time (*P* ≤ 0.05);

x-y Different letters in columns mean statistically significant differences between the group average, including the type of muscle (*P* ≤ 0.05); ±standard deviation.

WBC and WHC results for the refrigerated muscles showed that the mean values of these parameters were higher in the LM than in the BM (by 5.21% and 5.29%, respectively). The WHC and WBC determined for control samples were similar to the results obtained by (Kowalczyk et al., 2013) for muscles of White Koluda, hybrids of the White Koluda and Canada geese. Lower WHC values were obtained by (Biesiada-Drzazga, 2006; Wołoszyn et al., 2011) for chilled muscles of geese and different kinds of ducks.

In both types of muscles, it was found that with the extension of the frozen storage time, the WHC and WBC decreased (Table 2). It was shown that WHC for BM and WBC for both types of muscles were higher on the 30th and 90th day of their storage compared to the other analyzed periods. On the other hand, WHC in LM decreased significantly between the 30th and 90th day of storage in frozen conditions (Table 2). The lower WHC in muscles can be caused by autolytic transformations of cytoskeletal proteins occurring during frozen storage. The degradation of cytoskeletal proteins reduces the strength of the connections between myofibrils and the sarcolemma, facilitating the penetration of immobilized water into the intercellular space. Water from the intercellular spaces is then available as meat juice (Huff-Lonergan and Lonergan, 2005). The WHC and WBC values stated for both types of geese muscles agree with Farouk et al. (2004), Vieira et al. (2009), Leygonie et al. (2012b), Śmiecińska et al. (2015), Ablikim et al. (2016), and Wei et al. (2017) results. These authors analyzed changes of WBC and WHC in leg muscles of turkey, pork, lamb, and African ostrich stored from 1 to 12 months at −20°C and at −22°C.

As in case pH, significant (*P* ≤ 0.05) differences in WHC and WBC also occurred between muscle types, too (Table 2). It was shown that the breast muscles were characterized by lower values of the analyzed parameters throughout the frozen storage period than those obtained for the LM (Table 2). In addition, the highest difference in the value of the WHC and WBC parameter between the analyzed muscle types was found on 365th day of frozen storage (Table 2). The higher pH of the LM may have contributed to a slower rate of muscle protein denaturation. These muscles could bind more immobilized and added water (Huff-Lonergan and Lonergan, 2005). Thus, the LM can be characterized by a higher WHC and WBC compared to BM.

Cooking and defrosting losses are considered a critical quality indicator of frozen meat (Vieira et al., 2009; Songsaeng et al., 2010; Soyer et al., 2010; Leygonie et al., 2012a). The amount of DL depends, for example, on the degree of damage to the histological structure of muscle tissue and freezing denaturation of proteins responsible for WBC. As a result of freezing meat, its structure is loosened, which leads to a decrease in WHC during thawing and higher losses of meat juice during heat treatment (Lagerstedt et al., 2008; Muela et al., 2010). It was found that the values of CL from BM stored for 24 h in refrigeration condition were significantly higher (by 0.34%) than from LM (Table 2). CL in both types of muscles was higher (on average: 8%–10%) than the data given by Biesiada-Drzazga (2006), Gornowicz et al. (2012), and Damaziak et al. (2016) for geese muscles of the same genotype. The differences could result from short-term post-slaughter factors affecting the raw meat.

The CL and DL determined for both types of muscles increased with the length of their frozen storage time (Table 2). BM and LM stored for 270 and 365 d had higher DL (respectively: 1.60% and 1.46% on the 270th day and 1.87% and 1.54% on 365th day) and CL (respectively: 26.13% and 25.06% on day 270th and 26.65% and 25.74% on day 365th) compared to the other analyzed storage periods (Table 2). Also, Domaradzki et al. (2011), Lee et al. (2008), and Vieira et al. (2009) showed the value of CL and DL from chicken breast muscles, pork and beef meat, increased with the extension frozen storage time. In study of Ali and Zahran (2010) and Alonso et al. (2016), the rise in CL and DL from frozen meat was also influenced by changing pH. In our research, only on 365th day, the BM was characterized by a significantly higher DL compared to LM (Table 2). On the other hand, significant differences in CL of BM and LM were confirmed on the 180th day of frozen storage (by 1.55%). In the remaining frozen storage periods, the DL differences between the tested muscle types were smaller (by 0.08%–0.33%) and were not statistically confirmed (Table 2).

Summarized the changes in the functional properties of frozen geese muscles, it was found that with the changes in the pH value, their WHC and WBC decreased. From the 180th day, the muscles were characterized by reduced technological usefulness compared to the raw material stored for 24 h in refrigeration and freezing conditions for 30 and 90 d. The highest differences in CL between BM and LM (by 1.55%) were found on 180th day of frozen storage (Table 2). In all frozen storage time a slight DL from the analyzed types of muscles indicates that the freezing process was carried out optimally. The ice crystals formed inside only slightly damaged their tissue structure.

### Instrumental Analysis of Color

The changes in L*, a*, b*, C, h°, and ΔE during frozen storage of the tested muscles are presented in Table 3. BM stored 24 h were characterized by a significantly lower L* (by 2.20) and a higher a* value (by 0.83) compared to LM. In turn, the values of b* in the control samples of both types of muscles were similar, and the differences were not significant (*P* ≤ 0.05; Table 3).

**Table 3:** Color parameters (L*, a*, b*, C, h°) of the BM and LM of the White Kołuda geese.

Parameters	Type of muscle	Control group	Time of frozen storage (d)				
		C	30	90	180	270	365
		*n* = 18					
L*	BM	43.12^a^^,^^y^ ± 0.33	42.68^b^^,^^y^ ± 0.38	42.14^c^^,^^y^ ± 0.37	41.88^cd^^,^^y^ ± 0.52	41.54^d^^,^^y^ ± 0.48	40.79^e^^,^^y^ ± 0.75
	LM	45.32^a^^,^^x^ ± 0.65	44.53^b^^,^^x^ ± 0.43	44.19^bc^^,^^x^ ± 0.58	43.85^c^^,^^x^ ± 0.61	43.03^d^^,^^y^ ± 0.71	42.33^e^^,^^x^ ± 0.53
a*	BM	15.82^a^^,^^x^ ± 0.32	14.44^b^^,^^x^ ± 0.33	13.95^c^ ± 0.22	13.56^d^^,^^x^ ± 0.16	12.07^e^^,^^y^ ± 0.34	11.53^f^ ± 0.29
	LM	14.99^a^^,^^y^ ± 0.25	14.01^b^^,^^y^ ± 0.24	13.74^c^ ± 0.19	13.38^d^^,^^y^ ± 0.19	13.02^e^^,^^x^ ± 0.18	12.53^f^ ± 0.22
b*	BM	1.83^a^ ± 0.21	1.79^a^ ± 0.26	1.90^a^ ± 0.27	1.84^a^ ± 0.26	1.46^b^ ± 0.23	1.06^c^ ± 0.19
	LM	1.85^a^ ± 0.20	1.72^a^ ± 0.22	1.75^a^ ± 0.35	1.79^a^ ± 0.18	1.39^b^ ± 0.19	1.11^c^ ± 0.16
C	BM	15.93^a^^,^^x^ ± 0.32	14.55^b^^,^^x^ ± 0.30	14.08^c^ ± 0.29	13.68^d^ ± 0.17	12.16^e^^,^^y^ ± 0.32	11.58^f^ ± 0.23
	LM	15.10^a^^,^^y^ ± 0.19	14.12^b^^,^^y^ ± 0.19	13.85^c^ ± 0.28	13.50^d^ ± 0.22	13.09^e^^,^^x^ ± 0.11	11.58^f^ ± 0.26
h°	BM	6.60^b^ ± 0.48	7.07^a^ ± 0.72	7.76^a^ ± 1.03	7.73^a^ ± 1.02	6.90^ab^ ± 1.15	5.25^c^ ± 1.12
	LM	7.04^bc^ ± 1.09	7.00^a^ ± 0.83	7.26^a^ ± 1.10	7.62^a^ ± 0.97	6.09^c^ ± 1.07	5.06^d^ ± 1.37
ΔE between consecutive storage periods	BM	-	1.45^b^^,^^x^ ± 0.08	0.74^d^^,^^x^ ± 0.07	0.47^e^ ± 0.05	1.57^a^^,^^x^ ± 0.08	1.01^c^^,^^x^ ± 0.05
	LM	-	1.27^a^^,^^y^ ± 0.10	0.44^e^^,^^y^ ± 0.09	0.50^d^ ± 0.06	0.98^b^^,^^y^ ± 0.04	0.90^c^^,^^y^ ± 0.05
ΔE between raw and storage periods	BM	-	1.45^e^^,^^x^ ± 0.11	2.11^d^^,^^x^ ± 0.09	2.58^c^^,^^x^ ± 0.14	4.09^b^^,^^x^ ± 0.08	4.94^a^^,^^x^ ± 0.15
	LM	-	1.27^e^^,^^y^ ± 0.13	1.69^d^^,^^y^ ± 0.13	2.18^c^^,^^y^ ± 0.11	3.06^b^^,^^y^ ± 0.16	3.94^a^^,^^y^ ± 0.09

Abbreviations: BM, breast muscles; LM, leg muscles.

a-f Different letters in a row mean statistically significant differences between group average, including storage time (*P* ≤ 0.05);

x-y Different letters in columns mean statistically significant differences between the group average, including the type of muscle (*P* ≤ 0.05); ± standard deviation.

It was found that the color parameters L*, a*, and C of both tested types of muscles decreased with the extension frozen storage time (Table 3). The highest differences in L* parameter for BM were between the 270th and 365th and for LM between the 270th and 365th day of frozen storage. The highest differences in a* and C for LM (respectively: 0.49 and 1.51) occurred between the 270th and 365th and for BM (respectively: 1.49 and 1.52) between the 180th and 270th day of frozen storage. In the remaining periods, the differences in L*, a*, and C for BM and LM were lower. The decrease in L* and a* for both muscles during their frozen storage could be caused by the increasing share of MMb and the decreasing MbO_2_ in the total content of heme pigments. The b* and h° parameters for BM and LM varied depending on their frozen storage. Muscles stored for 30, 90, and 180 d were characterized by significantly higher values of b* and h° than those measured on the 270th and 365th day of storage (Table 3).

The highest difference in ΔE (1.57) was found between 180th and 270th day of frozen storage for BM. This means that the color changes between successive storage periods were small. In addition, consumers would not notice visible differences in meat color between storage periods in sensory evaluation. The highest differences in ΔE were for BM than LM during their frozen storage (Table 3), whereas ΔE between raw and storage periods was higher and in range 1.27 to 4.94. From 90th day of frozen storage, the differences were above 2.0 (Table 3). If the ΔE equaled more than 2.0, there is a real chance of occurring significant difference between these groups with traits analyzed in the paper. The value of 2.0 can therefore be considered as the value limiting noticeable changes in meat color (Haraf et al., 2009).

The values of L*, a*, and b* color parameters of both types of muscles are similar to the results obtained by Lee et al. (2008), Vieira et al. (2009), Ali and Zahran (2010), and Śmiecińska et al. (2015). These authors also found a decrease in the color parameters (L*, a*, b*) of turkey, chicken and beef meat stored for 6 to 12 months in freezing condition (−18°C and −28°C). Muela et al. (2010) explained the decrease in a* parameter of meat (along with the extension of its frozen storage time) by the progressing process of the so-called freeze denaturation of myoglobin. In turn, Zhang et al. (2005), Selani et al. (2011), Alonso et al. (2016), and Karwowska et al. (2017) found no significant changes in L*, a*, and b* of geese, pork, and beef muscles and during 7, 9, and 24 months of storage at −20°C and −24°C. As in our research, a significant decrease in C parameter for lamb and beef meat during frozen storage (for 60 and 90 d at −20°C) was found by Vieira et al. (2009) Kim et al. (2011, 2013). On the other hand, Zhang et al. (2005) and Leygonie et al. (2012b) did not note the effect of frozen storage of African ostrich and beef meat on changes in the C and h°.

Differences in L* and a* also occurred between the analyzed types of muscles (Table 3). The BM were characterized by lower values of L* in the analyzed storage periods compared to their values determined for the LM (Table 3). The differences in L* between the 2 types of muscles in the individual periods of their frozen storage could depend on the different content of heme pigments. It was also shown that a* during frozen storage depended on the type of muscle. BM stored for 30 d in freezing conditions were characterized by a higher intensity of red color than LM. In turn, LM was characterized by a higher value of this parameter measured only on the 270th day of their storage (by 0.95). However, there was no significant effect of the type of muscle on the values of C, h°, and b* determined for the analyzed frozen storage periods (Table 3).

The results of measurements of L* and a* obtained in own research are similar to Karwowska et al. (2017) results. These authors also found that a higher value characterized BM obtained from geese of the same genotype than those specified for LM.

In our previous study (Wereńska et al., 2022), in which there were the same materials, from the same delivery, it was shown that total heme pigment content and the MbO_2_ share decreased, whereas MMb increased in both types of muscles during frozen storage time. The obtained data indicate the progressing oxidation processes of MbO_2_, which leads to an increase in the relative content of MMb. The increase in the relative content of MMb leads to a decrease L* and a* parameter.

### Sensory Evaluation

The sensory evaluation of BM and LM stored for 24 h in refrigerated conditions showed that, the general appearance, color, smell, and consistency were similar for both types of geese muscles (Table 4). The results of the sensory assessment of color, general appearance, and smell obtained in our research are close to those obtained by Patsias et al. (2008) for chicken meat. Extending the frozen storage time of BM and LM lowered the scores of all sensory descriptors. The scores for the general appearance and smell were lower from the 180th day for BM and from the 90th day for LM frozen storage. It was also shown that a more favorable color characterized BM and LM on the 30th and 90th day than in the subsequent storage periods (Table 4). It should be noted that the values of all sensory descriptors for both types of muscles during the entire period of frozen storage were higher than 3 CU. According to the universal standards used in the sensory evaluation of meat, this meat will be considered tolerated by the consumer when deciding to purchase it (Miller, 2023).

**Table 4:** Sensory evaluation of BM and LM of White Kołuda geese.

Attributes (CU)	Type of muscle	Control group	Time of frozen storage (d)				
		C	30	90	180	270	365
		*n* = 18					
General appearance	BM	5.80^a^ ± 0.15	5.80^a^ ± 0.21	5.80^a^ ± 0.21	4.40^b^^,^^y^ ± 0.23	4.20^c^^,^^y^ ± 0.17	4.00^c^ ± 0.46
	LM	5.90^a^ ± 0.14	5.90^a^ ± 0.14	5.60^b^ ± 0.22	5.25^c^^,^^x^ ± 0.34	5.00^c^^,^^x^ ± 0.32	4.00^d^ ± 0.46
Color	BM	5.90^a^ ± 0.14	5.90^a^ ± 0.14	5.00^b^^,^^y^ ± 0.64	4.00^c^^,^^y^ ± 0.46	4.00^c^^,^^y^ ± 0.46	3.73^c^ ± 0.50
	LM	5.90^a^ ± 0.14	5.90^a^ ± 0.14	5.60^b^^,^^x^ ± 0.22	5.25^c^^,^^x^ ± 0.34	4.81^d^^,^^x^ ± 0.57	4.00^e^ ± 0.46
Smell	BM	5.90^a^ ± 0.14	5.88^a^ ± 0.21	5.80^a^ ± 0.21	5.25^b^ ± 0.50	4.00^c^ ± 0.46	4.00^c^ ± 0.46
	LM	5.90^a^ ± 0.14	5.92^a^ ± 0.18	5.60^b^ ± 0.22	5.25^c^ ± 0.34	4.00^d^ ± 0.46	4.00^d^ ± 0.46
Consistency	BM	5.92^a^ ± 0.18	5.90^a^ ± 0.14	5.88^a^ ± 0.21	5.10^b^^,^^y^ ± 0.64	4.08^c^ ± 0.58	4.03^c^ ± 0.72
	LM	5.90^a^ ± 0.14	5.90^a^ ± 0.14	5.92^a^ ± 0.18	5.50^b^^,^^x^ ± 0.51	4.15^c^ ± 0.58	4.13^c^ ± 0.61

Abbreviations: BM, breast muscles; LM, leg muscles.

a-e Different letters in a row mean statistically significant differences between group average, including storage time (*P* ≤ 0.05);

x-y Different letters in columns mean statistically significant differences between the group average, including the type of muscle (*P* ≤ 0.05); ± standard deviation.

Our results of sensory evaluation of BM and LM are similar to the effects of Klebaniuk et al. (2011) study. These authors also found a decrease in odor scores (intensity and desirability) of lamb meat stored for 6 months at −20°C. In turn, Brannan (2009) found no significant changes in the sensory evaluation of the color and smell of chicken meat stored only for 14 d at −18°C.

The factor differentiating changes in sensory parameters was a type of muscle, too. LM had higher scores for a general appearance at the 180th and 270th day, color at the 90th, 180th, 270th, and 365th day and consistency at the 180th day of their frozen storage than BM (Table 4).

## CONCLUSION

From the 180th day of frozen storage, the muscles were characterized by reduced technological usefulness compared to the raw material stored for 24 h in refrigeration and in freezing condition for 30 and 90 d. WHC and WBC decreased with extension of frozen storage time. Also when the goose meat was stored for 365 d at −20°C, unfavorable changes in its color occurred. These changes were also reflected in the decreased L* and a* of the BM and LM. The sensory analysis showed that from the 270th day of frozen storage, BM and LM were characterized by significantly lower scores for smell and consistency than muscles stored in cold storage and for 30, 90, and 180 d in freezing conditions. In addition, BM received lower scores for general appearance (on 180th and 270th day), L* value (on 90th, 180th, 270th, and 365th day), than LM. Changes in the quality of the frozen muscles occurred with different intensity, depending on their type. Therefore, it is difficult to clearly indicate which of them were characterized by more favorable values of the analyzed parameters. Moreover, it is hard to say, which type of muscles the changes determining the decrease in their quality were faster. Based on the analysis of changes in the examined qualitative characteristics, it can be suggested, that the optimal storage time for both types of muscle packed in heat-shrinkable foil at −20°C should not be longer than 180 d.

## DISCLOSURES

The authors declare no conflicts of interest in publication “impact of frozen storage on some functional properties and sensory evaluation of goose meat.”
